# Analysis of physical and physiological training demands in women’s basketball

**DOI:** 10.1371/journal.pone.0354357

**Published:** 2026-07-27

**Authors:** Pau Pons-Gomila, Julen Castellano, Julio Calleja-González, Javier Espasa-Labrador, Dimitrije Cabarkapa, Franc García

**Affiliations:** 1 Instituto Nacional de Educación Física de Catalunya (INEFC), Universidad de Barcelona (UB), Barcelona, España; 2 Futbol Club Barcelona, Área de Rendimiento Deportivo, Barcelona, España; 3 Departament of Physical Education and Sport, Faculty of Education and Sport, University of Basque Country (UPV/EHU), Vitoria, Spain; 4 Facultad de Kinesiología, Universidad de Zagreb, Zagreb, Croacia; 5 Jayhawk Athletic Performance Laboratory–Wu Tsai Human Performance Alliance, Department of Health, Sport and Exercise Sciences, University of Kansas, Lawrence, Kansas, United States of America; 6 D2 Lab, Novi Sad, Serbia; SARAH Network of Rehabilitation Hospitals: Rede SARAH de Hospitais de Reabilitacao, BRAZIL

## Abstract

**Purpose:**

The primary aim of the present study was to analyze the physical and physiological demands associated with different training tasks in women’s basketball.

**Methods:**

Fourteen basketball players were continuously monitored for 22 weeks using WIMU PRO^®^™ local positioning system and GARMIN^®^ heart rate monitors. The variables examined included: distance (DIST in m), number of accelerations (>2 m·s^-2^, HACC) and decelerations (<−2 m·s^-2^, HDEC); high speed running (>18 km·h^-1^, HSR in m), number of jumps (JUMPS), player load (PL in arbitrary units) and heart rate zones (>80% of maximum heart rate, HRZ in seconds). Training tasks were classified into three orientations: directed-oriented (DIR, drills without opposition), special-oriented (SPE, small-sided games), and competitive-oriented (COM, 4-on-4 and 5-on-5 formats); and further grouped by three levels of court space. The Shapiro–Wilk test was used to verify data normality. Linear mixed-effects models were used to analyze all variables. Skewed variables were log-transformed to meet model assumptions. Significance was set at p < 0.05.

**Results:**

The SPE tasks showed higher values than COM tasks (p < 0.001) in all variables except for HRZ. Also, SPE tasks reported greater values than DIR in the following variables: HRZ and HSR (both p < 0.001) and PL (p = 0.008). Finally, DIR tasks showed higher values than COM tasks in DIST, HACC, HDEC and JUMPS (all p < 0.001), whereas COM tasks exhibited higher values than DIR tasks in HRZ (p < 0.001). Considering the court space in which the tasks were performed, differences were found in all the variables analyzed, with larger spaces generally resulting in greater demands in most variables.

**Conclusions:**

The results describe that task orientation and court space influence the load demands in women’s basketball. DIR and SPE tasks elicited greater intensities than COM tasks, suggesting that training design should be strategically adapted according to the desired physical and physiological stimuli.

## 1 Introduction

Advances in microtechnology have profoundly transformed performance analysis and decision-making processes in team sports such as basketball [1]. The development of smaller, precise, and highly integrated devices now enables the collection of large volumes of data during both training and competition [2]. Such data can provide strength and conditioning coaches and technical staff with valuable insights to optimize on-court athletic performance. Moreover, the detailed information obtained through these technologies facilitates evidence-based planning grounded in real team data. This approach is particularly important in team sports, as it supports informed decision making, enhances individual player development and collective tactical efficiency, and allows for more precise training periodization [3].

In particular, basketball is a high-intensity intermittent sport, characterized by both aerobic and anaerobic demands, involving frequent changes of direction, accelerations and decelerations, jumps, sprints, and sport-specific technical actions [3]. These demands occur within a highly dynamic and unpredictable environment, often accompanied by physical contact, making basketball one of the most physically and cognitively demanding team sports [4]. Consequently, quantifying training load represents a fundamental strategy for sports practitioners to monitor athletes’ responses to training and competition. This approach not only helps reduce injury risk [5] but also improves player availability, as a key to sustaining team consistency and maximizing performance across the full competitive season span [6].

In this context, training load can be broadly categorized into external load (EL) and internal load (IL) [2,7,8]. Specifically, EL refers to the physical demands imposed on players, representing the mechanical and locomotor stimuli experienced during training and competition [8,9]. In contrast, IL reflects the physiological and psychological responses to the aforementioned stimuli [2]. Also, the introduction of global positioning systems (GPS) and inertial measurement devices has revolutionized performance monitoring in high-performance sport, allowing for a more comprehensive understanding of the physical and technical demands associated with various activities [10]. However, in indoor sports such as basketball, where precise movement tracking is essential, GPS systems are often compromised by environmental interference, reducing positional accuracy and data reliability [11]. To address this limitation, local positioning systems (LPS) based on ultra-wideband technology have been developed, along with devices integrating accelerometers, gyroscopes, and magnetometers [2]. Complementary to these systems, physiological monitoring technologies enable the assessment of athletes’ responses to training and competition stimuli [12]. Among these, heart rate monitoring remains one of the most widely used methods to evaluate training task intensity, given its strong association with maximal oxygen uptake (VO_2_max) and its utility in quantifying overall physiological load [13].

Understanding how training tasks to which athletes are exposed affect them could be a key aspect to ensure that training matches with the physical and physiological demands of competition [14]. In relation to this, it is essential to analyze training tasks to identify the ones that are more appropriate to induce adaptations that improve athletes’ performance [15]. Particularly in female basketball, differences in total distance have been observed among teams with different playing styles [16], and it has also been confirmed that physiological demands vary according to competitive level and playing positions in female basketball players [14,17].

Comparing training and competition loads provides valuable insight into whether the demands imposed during practice adequately replicate those experienced in actual game-like scenarios. Training loads might exceed competition demands [18]. Therefore, effective training periodization is essential to ensure athletes reach optimal performance during competition, while minimizing fatigue-related performance decrements [19]. Yet, the design of basketball training sessions depends on several contextual factors, including team characteristics, coaching philosophy, and competitive objectives. However, given the complexity of the sport, establishing a structured and progressive approach to training organization is crucial [20]. In this context, Schelling et al. [19] proposed a methodological framework based on the progression of task specificity, distinguishing four orientations: (i) general-oriented tasks (i.e., focused on developing various strength qualities with different movement speeds and ranges of motion not directly related to basketball-specific techniques); (ii) directed-oriented tasks (i.e., basketball-specific drills characterized by high intensity actions, simple decision making requirements, and the absence of opposition); (iii) special-oriented tasks (i.e., consisting of small-sided games that integrate decision-making and tactical execution, such as 2-on-2, 3-on-3, and 4-on-3); and (iv) competitive-oriented tasks (i.e., highest level of specificity by combining cognitive, physical, and physiological demands characteristic of actual competition, such as 5-on-5 and 4-on-4).

Expanding upon this framework, several studies have utilized physical and physiological metrics to examine how training tasks with varying degrees of opposition influence overall task demands and athlete responses [21–29]. Based on these research reports, the most frequently analyzed variables include total distance covered and a number of accelerations and decelerations [21–23], high-speed running [24], jumps [26], player load [27] and heart rate zones [30]. Collectively, these parameters provide valuable insights into how different practice conditions alter the physical and physiological demands placed on athletes.

Given the scarcity of studies about woman’s basketball using LPS [28,31,32], the present study aims to describe the demands imposed on women basketball players in terms of both task orientation and the spatial dimensions in which these tasks are performed, in order to provide a more comprehensive understanding of training demands in women’s basketball. The findings may help refine planning, monitoring, and injury prevention strategies, offering insights that extend beyond those reported in male basketball populations.

## 2 Materials and methods

### 2.1 Design

The present investigation followed a descriptive longitudinal design and was conducted on a women’s basketball team competing in the Spanish 2^nd^ Division (Liga Femenina Challenge). A total of 37 training sessions were monitored over a 22 week competitive period (October 2024 through March 2025). The sample comprised all available players from the team during the monitoring period. Given the observational nature of the study and its implementation in a real-world basketball setting, no a priori sample size calculation was performed. Despite including only 14 players, the longitudinal design generated a substantial dataset comprising 148 training tasks, and an average of 101 task records per player. Furthermore, the use of linear mixed models allowed all repeated observations to be considered while accounting for within-player variability.

Prior to data collection, players were thoroughly familiarized with all monitoring procedures through several introductory sessions. On average, each athlete participated in 24.9 ± 9.1 training sessions. All practices were conducted from Monday through Friday, beginning at either 9:00 h or 10:00 h.

### 2.2 Participants

Fourteen adult female basketball players (age = 25.8 ± 3.4 years, height = 177.6 ± 8.0 cm, body mass = 72.6 ± 10.5 kg) volunteered to participate in the present study, with all of them playing for a 2nd division team in Spain (experience at this level = 1.8 ± 1.2 years). Based on the classification by Mckay et al. [33], players were considered highly trained athletes competing at the national level.

Before the start of the study, all players underwent routine physical evaluations conducted by the club’s medical staff as part of their standard preseason assessments. Players were included in the analysis if they completed the entire training session and if all variables were successfully recorded by the inertial and LPS sensors, whereas players were excluded if they failed to complete a session or sustained an injury that prevented further participation.

Before the study, all players were informed about the research procedures and voluntarily provided written consent to participate. The study was approved by the Ethics Committee of Clinical Research of the Sports Administration of Catalonia (013/CEIGC/2022) and conducted in accordance with the ethical standards for biomedical research involving human subjects outlined in the Declaration of Helsinki [34].

### 2.3 Variables

In line with previous research [21–27,30], the dependent variables included in the analysis are presented in Table 1. All variables were normalized per minute to allow comparisons between training tasks of different durations.

**Table 1:** Abbreviations, units, and description of the selected variables.

Variable	Abbreviation	Units	Description
Distance	DIST	m·min^-1^	Distance covered in meters
Accelerations	HACC	n·min^-1^	Number of accelerations above 2 m·s^-2^
Decelerations	HDEC	n·min^-1^	Number of decelerations above −2 m·s^-2^
High speed running	HSR	n·min^-1^	Number of times exceeding 18 km·h^-1^
Jumps	JUMPS	n·min^-1^	Number of jumps performed
Player load	PL	au·min^-1^	Sum of squared changes in acceleration across the three vectors (x, y, z) divided by 100
HR zone	HRZ	s·min^-1^	Time spent >80% of maximum heart rate

(HR) - heart rate; (au) - arbitrary units.

### 2.4 Training tasks

To analyze training tasks, three main groups were defined according to their orientation [19,35]. Although general-oriented tasks are recognized in the training task classification framework, they were excluded from the present study because they are typically performed outside the basketball court and are not directly associated with basketball-specific practice situations. Given that the purpose of this study was to analyze the physical and physiological demands of on-court basketball training, only directed, special, and competitive-oriented tasks were considered. Within each orientation, tasks were further categorized based on the court space, grouped into three categories: (i) tasks performed on a ½ or ¾ court, (ii) tasks performed across 1-2 courts, and (iii) tasks performed on a ½ court combined with 2 or more courts, as presented in Table 2. Also, it should be noted that warm-up activities, individual training drills (1-on-0), and SPE tasks involving 1-on-1, 2-on-1, or 2-on-2 formats were excluded from the analysis, as these occurred infrequently and could introduce bias into the results.

**Table 2:** Description of task groups according to both orientation, court space, and duration.

Orientation	Players per team		Court space	Abbreviation	Average duration (min)
DIR	3-on-0, 4-on-0, 5-on-0, 4-on-0/3-on-0	1	½ court or ¾ court	DIR1	8.0 ± 7.2
		2	1 and 2 courts	DIR2	9.9 ± 3.7
		3	½ + 2 or more courts	DIR3	10.8 ± 0.3
SPE	3-on-2, 3-on-3, 4-on-2, 4-on-3, 3-on-3/3-on-2, 3-on-2/3-on-3/4-on-3, 3-on-4/ 4-on-3	1	½ court or ¾ court	SPE1	14.6 ± 8.0
		2	1 and 2 courts	SPE2	11.7 ± 5.0
		3	½ + 2 or more courts	SPE3	11.2 ± 4.5
COM	4-on-4, 5-on-4, 5-on-5, 4-on-4/5-on-4	1	½ court or ¾ court	COM1	16.0 ± 6.9
		2	1 and 2 courts	COM2	14.4 ± 6.6
		3	½ + 2 or more courts	COM3	19.8 ± 9.7

(x-on-x) - indicates the number of players per team.

### 2.5 Procedures

All monitored training sessions were conducted on the same official court in similar environmental conditions. Practices were scheduled from Monday through Friday with one rest day each week. Athletes were equipped with a GARMIN™ HR band (Garmin Ltd^®^., Olathe, KS, USA) for heart rate monitoring, and their movements were recorded using WIMU PRO™ inertial devices (Realtrack Systems SL^®^, Almeria, Spain). Each unit incorporated four triaxial accelerometers (100 Hz), a gyroscope (8000°/s), a magnetometer (100 Hz) and an ultra-wideband local positioning system (18 Hz). The devices were secured in a specialized WIMU™ vest positioned on the upper back to minimize sensor displacement. Prior to each session, all units were automatically calibrated using the WIMU™ autonomous calibration system per manufacturer guidelines [36]. All athletes were familiarized with the equipment during the week preceding the start of the monitoring process. The WIMU PRO™ devices have been validated for accuracy when using the ultra-wideband positioning system [37]. This system employs six antennas placed 12 m outside the court boundaries, with three antennas positioned along each baseline and spaced 17 m apart. This configuration forms a rectangular network that fully encloses the court, ensuring complete signal coverage and minimizing data loss. The antennas were mounted 7 m above the ground and connected, calibrated, and synchronized in accordance with the manufacturer’s specifications [22]. The monitoring data were downloaded and analyzed using the manufacturer’s specific software SPRO™ (Version 950, RealTrack Systems^®^, Almeria, Spain).

### 2.6 Statistical analysis

Data normality was assessed using the Shapiro-Wilk test [38]. In addition, distributional assumptions were visually inspected using Q-Q plots and histograms. Homoscedasticity was evaluated through graphical examination of residuals and variance patterns. Variables showing clear departures from normality or heteroscedasticity were log-transformed prior to inferential analysis to improve distributional symmetry and variance homogeneity [39].

Data were summarized as estimated marginal means with 95% confidence intervals (CI). All dependent variables were analyzed using linear mixed-effects models. The models were used to examine differences between task categories based on the orientations (directed, special, and competitive) and the court-space. These factors were included as fixed effects in the models. Players were included as random effects to account for repeated measurements within the same individual across training sessions. This modelling approach allowed all available data to be retained in the analysis while appropriately accounting for the non-independence of observations. Skewed dependent variables (HACC, HDEC, HSR, HRZ, and JUMPS) were natural log-transformed to improve normality and homoscedasticity. Residual Q-Q plots and histograms confirmed that model assumptions were adequately met after transformation. Bonferroni-adjusted post-hoc comparisons were performed when significant interaction effects were present. To quantify the magnitude of between-condition differences, standardized effect sizes (Cohen’s d) were calculated from the mixed-model estimates. Effect sizes were interpreted as trivial (<0.20), small (0.20–0.59), moderate (0.6–1.19), large (1.20–1.99), and very large (≥2.00) [40]. For log-transformed variables, fixed effect estimates were back transformed by exponentiation to facilitate interpretation in the original measurement scale. Results were expressed as proportional or percentage differences, allowing effects to be interpreted in practical terms. This approach is appropriate for positively skewed variables and facilitates meaningful interpretation of changes in training demands. Statistical significance was set p < 0.05. All analyses were conducted using Jamovi (Version 2.7.12; Jamovi Project, 2025).

## 3 Results

**Fig 1:**
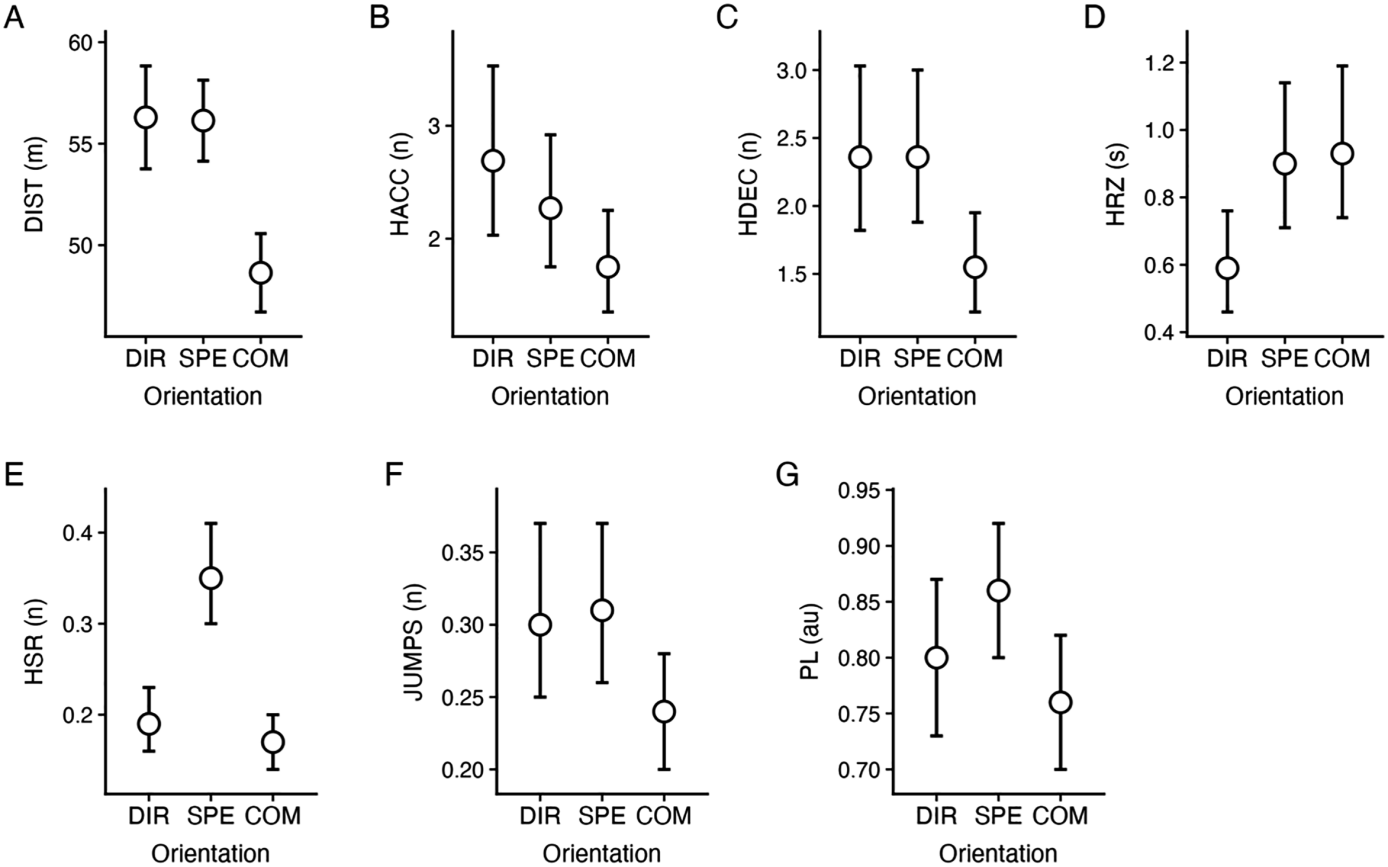
Estimated marginal means (±95% CI) for physical and physiological variables according to task orientation. (COM) - competitive-oriented tasks; (DIR) - directed-oriented tasks; (SPE) - special-oriented tasks. Values were derived from the linear mixed-effects models.

Results are presented in Tables 3-5 and in Fig 1. In the analysis by task orientation, statistically significant differences were observed between DIR and COM tasks in DIST, HACC, HDEC, HRZ, and JUMPS (all p < 0.001). For DIST, a small effect size was observed (d = 0.47). For log-transformed variables, DIR tasks exhibited higher values than COM tasks for HACC, HDEC, and JUMPS with proportional differences ranging from approximately 22–35%. In contrast, HRZ showed higher values during COM tasks compared with DIR tasks, with an increase of approximately 58%. Comparisons between DIR and SPE tasks revealed significant differences in HRZ and HSR (both p < 0.001) as well as in PL (p = 0.008). The difference in PL was associated with a small effect size (d = 0.24), whereas HRZ and HSR exhibited proportional differences indicative of higher demands during SPE tasks. Between SPE and COM tasks, statistically significant differences were observed in all variables (p < 0.001) except for HRZ. SPE tasks elicited higher values than COM tasks for all log-transformed variables, with proportional increases ranging from approximately 22–52%. Small effect sizes were also observed for DIST (d = 0.46) and PL (d= 0.40).

**Table 3:** Estimated marginal means and 95% confidence intervals (CI) according to task orientation and court space for each variable.

Variable (unit)	Orientation	Court space	Mean	95% CI	
				Lower	Upper
**DIST (m)**	COM	COM1	39.81	37.65	41.98
		COM2	54.72	52.07	57.38
		COM3	62.55	59.81	65.29
	DIR	DIR1	53.79	50.21	57.38
		DIR2	57.15	54.38	59.93
		DIR3	59.21	53.11	65.30
	SPE	SPE1	44.37	41.83	46.91
		SPE2	59.78	57.51	62.05
		SPE3	68.33	65.09	71.58
**HACC (n)**	COM	COM1	1.21	0.96	1.54
		COM2	2.23	1.72	2.89
		COM3	3.25	2.48	4.22
	DIR	DIR1	3.13	2.27	4.31
		DIR2	2.34	1.79	3.03
		DIR3	4.71	2.97	7.39
	SPE	SPE1	1.07	0.84	1.38
		SPE2	2.86	2.25	3.63
		SPE3	5.26	3.94	7.03
**HDEC (n)**	COM	COM1	1.02	0.81	1.28
		COM2	2.05	1.62	2.64
		COM3	3.00	2.32	3.86
	DIR	DIR1	2.80	2.05	3.82
		DIR2	2.01	1.57	2.61
		DIR3	4.22	2.75	6.49
	SPE	SPE1	1.17	0.91	1.49
		SPE2	2.92	2.29	3.67
		SPE3	5.10	3.86	6.75
**HRZ (s)**	COM	COM1	0.70	0.55	0.90
		COM2	1.15	0.89	1.49
		COM3	1.39	1.07	1.82
	DIR	DIR1	0.78	0.57	1.06
		DIR2	0.45	0.35	0.60
		DIR3	1.08	0.73	1.62
	SPE	SPE1	0.67	0.52	0.87
		SPE2	0.97	0.76	1.25
		SPE3	1.26	0.94	1.67
**HSR (n)**	COM	COM1	0.05	0.03	0.09
		COM2	0.18	0.15	0.22
		COM3	0.17	0.14	0.20
	DIR	DIR1	0.15	0.09	0.23
		DIR2	0.21	0.17	0.26
		DIR3	0.15	0.11	0.22
	SPE	SPE1	0.31	0.23	0.42
		SPE2	0.34	0.29	0.40
		SPE3	0.40	0.33	0.49
**JUMPS (n)**	COM	COM1	0.23	0.19	0.27
		COM2	0.24	0.20	0.30
		COM3	0.25	0.21	0.30
	DIR	DIR1	0.54	0.43	0.68
		DIR2	0.24	0.19	0.29
		DIR3	0.24	0.17	0.32
	SPE	SPE1	0.28	0.23	0.33
		SPE2	0.32	0.27	0.38
		SPE3	0.34	0.28	0.42
**PL (au)**	COM	COM1	0.64	0.58	0.70
		COM2	0.84	0.77	0.91
		COM3	0.96	0.89	1.03
	DIR	DIR1	0.83	0.75	0.91
		DIR2	0.78	0.71	0.85
		DIR3	0.86	0.75	0.97
	SPE	SPE1	0.71	0.64	0.78
		SPE2	0.90	0.83	0.96
		SPE3	1.04	0.97	1.12

Values are presented as estimated marginal means with 95%CI derived from linear mixed-effects models. Orientation refers to the type of task performed (COM = competitive; DIR = directed, SPE = special). Court space refers to the playing area used in the task (1 = ½ or ¾ court, 2 = between 1 and 2 courts, 3 = ½ + 2 or more courts). Estimated marginal means represent the orientation x court space combinations. Variable units are indicated in parentheses.

**Table 4:** Comparisons between task orientations for each variable.

Variable (unit)	Comparison	p-value	Effect size	95% CI	
				Lower	Upper
**DIST (m)**	COM vs DIR	<.001*	d = -0.47 (small)	−0.63	−0.32
	COM vs SPE	<.001*	d = -0.46 (small)	−0.58	−0.35
	DIR vs SPE	1.000	d = 0.01 (trivial)	−0.15	0.17
**HACC (n)**	COM vs DIR	<.001*	−34.95%	−50.6%	−19.3%
	COM vs SPE	<.001*	−22.89%	−34.7%	−11.1%
	DIR vs SPE	0.145	18.53%	0.9%	36.2%
**HDEC (n)**	COM vs DIR	<.001*	−34.30%	−50.0%	−18.6%
	COM vs SPE	<.001*	−34.95%	−46.7%	−23.2%
	DIR vs SPE	1.000	0.00%	−15.7%	15.7%
**HRZ (s)**	COM vs DIR	<.001*	58.41%	44.7%	72.1%
	COM vs SPE	1.000	4.08%	−5.7%	13.9%
	DIR vs SPE	<.001*	−34.30%	−50.0%	−18.6%
**HSR (n)**	COM vs DIR	0.253	−13.06%	−28.7%	2.6%
	COM vs SPE	<.001*	−52.29%	−64.0%	−40.5%
	DIR vs SPE	<.001*	−45.12%	−60.8%	−29.4%
**JUMPS (n)**	COM vs DIR	<.001*	−22.12%	−31.9%	−12.3%
	COM vs SPE	<.001*	−22.89%	−30.7%	−15.1%
	DIR vs SPE	1.000	−1.00%	−10.8%	8.8%
**PL (au)**	COM vs DIR	0.115	d = -0.16 (trivial)	−0.32	0.00
	COM vs SPE	<.001*	d = -0.40 (small)	−0.48	−0.32
	DIR vs SPE	0.008*	d = -0.24(small)	−0.40	−0.08

Pairwise comparisons between task orientations were conducted using post-hoc tests derived from linear mixed-effects models. Values are presented as p-values, effect sizes, and 95% CI. For normally distributed variables, effect size is expressed as Cohen’s d and interpreted as trivial (<0.20), small (0.20–0.59), moderate (0.60–1.19), large (1.20–1.99) and very large (≥2.00). For log-transformed variables, effects are expressed as percentage differences with corresponding 95% CI. Asterisk (*) indicates statistical significance (p < 0.05). Negative values indicate lower values in the first-named condition of each comparison.

**Table 5:** Comparisons between court space conditions within each task orientations for each variable.

Variable (unit)	Comparison	p-value	Effect size	95% CI	
				Lower	Upper
**DIST (m)**	COM1 vs COM2	<.001*	d = -1.08(moderate)	−1.27	−0.89
	COM1 vs COM3	<.001*	d = -1.64 (large)	−1.84	−1.45
	COM2 vs COM3	<.001*	d = -0.57 (small)	−0.79	−0.34
	DIR1 vs DIR2	1.000	d = -0.24 (small)	−0.53	0.04
	DIR1 vs DIR3	1.000	d = -0.39 (small)	−0.88	0.10
	DIR2 vs DIR3	1.000	d = -0.15 (trivial)	−0.61	0.31
	SPE1 vs SPE2	<.001*	d = -1.12 (moderate)	−1.31	−0.93
	SPE1 vs SPE3	<.001*	d = -1.74 (large)	−1.99	−1.49
	SPE2 vs SPE3	<.001*	d = -0.62 (moderate)	−0.86	−0.38
**HACC (n)**	COM1 vs COM2	<.001*	−45.66%	−63.3%	−28.0%
	COM1 vs COM3	<.001*	−62.47%	−82.1%	−42.9%
	COM2 vs COM3	0.027*	−31.61%	−53.2%	−10.1%
	DIR1 vs DIR2	1.000	33.64%	4.2%	63.0%
	DIR1 vs DIR3	1.000	−33.63%	−80.7%	13.4%
	DIR2 vs DIR3	0.063	−50.34%	−93.5%	−7.2%
	SPE1 vs SPE2	<.001*	−62.47%	−80.1%	−44.8%
	SPE1 vs SPE3	<.001*	−79.61%	−103.1%	−56.1%
	SPE2 vs SPE3	<.001*	−45.66%	−69.2%	−22.1%
**HDEC (n)**	COM1 vs COM2	<.001*	−50.84%	−68.5%	−33.2%
	COM1 vs COM3	<.001*	−66.04%	−83.7%	−48.4%
	COM2 vs COM3	0.015*	−30.93%	−52.5%	−9.4%
	DIR1 vs DIR2	0.859	39.10%	11.7%	66.5%
	DIR1 vs DIR3	1.000	−33.63%	−78.7%	11.4%
	DIR2 vs DIR3	0.02*	−52.29%	−93.4%	−11.1%
	SPE1 vs SPE2	<.001*	−59.75%	−77.4%	−42.1%
	SPE1 vs SPE3	<.001*	−77.01%	−100.5%	−53.5%
	SPE2 vs SPE3	<.001*	−42.88%	−64.4%	−21.3%
**HRZ (s)**	COM1 vs COM2	<.001*	−38.74%	−54.4%	−23.1%
	COM1 vs COM3	<.001*	−49.84%	−67.5%	−32.2%
	COM2 vs COM3	1.000	−17.30%	−36.9%	2.3%
	DIR1 vs DIR2	0.004*	71.60%	44.2%	99.0%
	DIR1 vs DIR3	1.000	−28.11%	−69.3%	13.1%
	DIR2 vs DIR3	<.001*	−58.10%	−95.3%	−20.9%
	SPE1 vs SPE2	<.001*	−30.93%	−46.6%	−15.2%
	SPE1 vs SPE3	<.001*	−46.74%	−68.3%	−25.2%
	SPE2 vs SPE3	0.589	−22.12%	−43.7%	−0.6%
**HSR (n)**	COM1 vs COM2	<.001*	−71.06%	−127.9%	−14.2%
	COM1 vs COM3	0.003*	−68.02%	−124.9%	−11.2%
	COM2 vs COM3	1.000	10.52%	−7.1%	28.2%
	DIR1 vs DIR2	1.000	−30.23%	−75.3%	14.8%
	DIR1 vs DIR3	1.000	−4.88%	−59.8%	50.0%
	DIR2 vs DIR3	1.000	36.34%	−0.9%	73.6%
	SPE1 vs SPE2	1.000	−9.52%	−38.9%	19.9%
	SPE1 vs SPE3	1.000	−22.89%	−54.3%	8.5%
	SPE2 vs SPE3	1.000	−14.79%	−32.4%	2.9%
**JUMPS (n)**	COM1 vs COM2	1.000	−5.82%	−17.6%	5.9%
	COM1 vs COM3	1.000	−8.61%	−20.4%	3.2%
	COM2 vs COM3	1.000	−2.96%	−16.7%	10.8%
	DIR1 vs DIR2	<.001*	129.33%	109.7%	148.9%
	DIR1 vs DIR3	<.001*	129.33%	98.0%	160.7%
	DIR2 vs DIR3	1.000	0.00%	−29.4%	29.4%
	SPE1 vs SPE2	0.342	−14.79%	−26.5%	−3.0%
	SPE1 vs SPE3	0.287	−19.75%	−35.4%	−4.1%
	SPE2 vs SPE3	1.000	−5.82%	−21.5%	9.9%
**PL (au)**	COM1 vs COM2	<.001*	d = -0.91(moderate)	−1.09	−0.73
	COM1 vs COM3	<.001*	d = -1.45(large)	−1.63	−1.28
	COM2 vs COM3	<.001*	d = -0.55(small)	−0.81	−0.28
	DIR1 vs DIR2	1.000	d = 0.23(small)	−0.04	0.49
	DIR1 vs DIR3	1.000	d = -0.18(trivial)	−0.72	0.35
	DIR2 vs DIR3	1.000	d = -0.41(small)	−0.85	0.04
	SPE1 vs SPE2	<.001*	d = -0.86(moderate)	−1.04	−0.69
	SPE1 vs SPE3	<.001*	d = -1.50(large)	−1.77	−1.23
	SPE2 vs SPE3	<.001*	d = -0.64(moderate)	−0.90	−0.37

Pairwise comparisons between court space conditions within each task orientations were conducted using post-hoc tests derived from linear mixed-effects models. Values are presented as p-values, effect sizes and 95% CI. For normally distributed variables, effect size is expressed as Cohen’s d and interpreted as trivial (<0.20), small (0.20–0.59), moderate (0.60–1.19), large (1.20–1.99) and very large (≥2.00). For log-transformed variables, effects are expressed as percentage differences with corresponding 95% CI. Asterisk (*) indicates statistical significance (p < 0.05). Negative values indicate lower values in the first-named condition of each comparison.

When classifying task orientations into three levels according to the court space (Table 2), significant differences were observed for DIST in SPE and COM tasks (p < 0.001). In SPE tasks, larger court-spaces were associated with higher DIST values, with effect sizes ranging from moderate to large (d = 0.62–1.74). Similarly, COM tasks showed higher DIST values as court space increased, with effect sizes ranging from small to large (d = 0.57–1.45). The variables HACC and HDEC showed significant differences across court space levels in SPE and COM tasks (p < 0.027–0.001). In both task orientations, larger playing spaces were associated with higher values, with proportional increases ranging from approximately 30–80%, indicating a clear progressive increase from smaller to larger court spaces in both SPE and COM. Significant differences were also observed for HRZ across court space levels within all task orientations (p < 0.004–0.001). Larger playing spaces were associated with higher HRZ values, with proportional increases ranging from approximately 30–70%. HSR showed significant differences in COM tasks across court space levels (p < 0.003–0.001). Tasks performed between 1 and 2 courts and ½ + 2 or more courts elicited substantially higher HSR values compared with tasks performed in the first court space level (½ court or ¾) with proportional increases of approximately 68–71%. Regarding JUMPS, differences were observed in DIR tasks at different space levels (p < 0.001) with tasks performed in ½ or ¾ court showing substantially higher values than those performed on the other court space levels with a proportional increase of approximately 129%. Additionally, PL differed significantly across court space levels in SPE and COM tasks (p < 0.001), with higher values consistently observed in the largest playing spaces. Effect sizes ranged from moderate to large in SPE (d = 0.64–1.50) and from small to large in COM (d = 0.28–1.28).

## 4 Discussion

The primary objective of this study was to examine the physical demands and physiological responses of female basketball players during training tasks, considering both the orientation and the court space in which they were performed. To the best of the authors’ knowledge, this is the first study to analyze the demands and responses of women’s basketball training tasks using LPS technology. The main findings indicate that task orientation and court space substantially influence player’s physical demands and physiological responseas DIR and SPE tasks generally resulted in higher intensities than COM tasks, larger spaces tended to impose greater demands, and SPE and COM showed a consistent space-load relationship while DIR occasionally produced higher intensity in reduced areas. Overall, these results highlight the importance of understanding how specific training tasks affect players’ workloads to determine the most appropriate timing and context for their implementation within a session or microcycle.

It is important to note that the majority of previously published research reports addressing this topic have been conducted on professional or semi-professional male basketball players, resulting in a relative lack of data on female athletes. Some investigations comparing competition demands between male and female atheltes have reported that, although certain differences exist, they are not necessarily substantial and often depend on contextual factors such as playing position [41]. Overall, research indicates that female players tend to cover less total distance and engage in fewer high-intensity actions compared with their male counterparts, which has been largely attributed to anthropometric and physiological differences [16,42]. Interestingly, Scanlan et al. [43] observed that female basketball players exhibited greater running demands, whereas male basketball players performed more dribbling actions. Yet, no significant sex-specific differences were noted in total distance covered, sprinting frequency, as well as overall intermittent demands [43]. In addition to sex-related differences, playing position may also influence the physical and physiological demands experienced by basketball players. Previous studies have reported position-specific variations in locomotor activity, high-intensity actions, and physiological responses, with guards, forwards, and centers often exhibiting distinct activity profiles during both training and competition [15,22]. Therefore, although the present study focused on examining the overall team demands, the responses observed may not be uniform across all playing positions, and future investigations should examine whether task orientation and court space manipulation affect positional groups differently.

Task orientation represents one of the main factors influencing the physical demand and physiological response of training tasks [19]. Previous research has identified differences in total load among tasks with varying orientations, showing that DIR tasks can elicit relatively high loads despite being unopposed and of lower tactical complexity [44,45]. Such, these tasks are frequently incorporated into the activation phase (warm-up time) of training sessions in senior teams, as they allow for tactical rehearsal and refinement of isolated game situations [46]. Although the cognitive load associated with such drills is typically low [44], the findings of the present study indicate that they still impose substantial physical demands.

Expanding on these observations, the results of the present investigation align with previous research reports [19,47], which indicate that SPE tasks, commonly defined as small-sided games or superiority/inferiority situations, are highly demanding from a physical standpoint. Sansone et al. [47] noted that the inclusion of defenders serves as a tactical constraint that increases movement intensity and overall workload. Consistent with these findings, SPE tasks in the present study generally elicited higher physical and physiological demands than COM tasks for the majority of the variables analyzed, suggesting that these drills impose a greater overall load on the athletes. Similar results have been reported in studies with male players. For example, Sosa et al. [29] found that tasks performed in larger spaces with fewer defenders tend to elicit greater physiological demands, whereas smaller, more crowded spaces are associated with higher physical intensity. Among these, SPE formats such as 3-on-3 appear particularly demanding due to the balance they strike between space, opposition, and task complexity. Collectively, these findings underscore the importance of manipulating task design to optimize conditioning while maintaining tactical representativeness. Additionally, the observation that SPE tasks were more demanding than COM tasks across nearly all variables suggests that they may serve as a particularly effective stimulus for enhancing players’ physical capacities.

In a high-performance context, the tactical component plays a central role, requiring substantial time to train game-realistic situations involving several players (4-on-4 or 5-on-5) that closely replicate competitive scenarios and promote contextual adaptations [48]. These competition-like game situations often involve multiple players operating within reduced spaces, demanding rapid decision-making to create tactical advantages, and thereby generating a high cognitive load [19]. While COM tasks remain essential for replicating the tactical and cognitive demands of real-game contexts [48], SPE drills appear to provide superior conditioning benefits by imposing greater physiological and mechanical stress [49]. Thus, strategically integrating these tasks within the training microcycle could accelerate specific physical adaptations, particularly those related to high-intensity actions such as accelerations, decelerations, and high-speed running. In this regard, SPE tasks can be viewed as a complementary tool to COM tasks, supporting physical development while preserving a high degree of tactical representativeness. However, it should be noted that the elevated physical and physiological stress associated with these tasks may also increase the risk of non-functional overreaching or acute injury if not properly managed [50]. Accordingly, careful load monitoring and well-structured periodization are crucial to optimize their benefits while minimizing potential adverse effects. From an applied perspective, the observed differences between task orientations may assist coaches in planning and regulating training load throughout the competitive season. Specifically, SPE tasks may be strategically implemented during sessions that aim to expose players to greater physical and physiological demands, whereas DIR tasks may be more appropriate during activation phases or technical-focused sessions requiring lower cognitive demands. COM tasks, in turn, remain particularly useful when the objective is to reproduce the tactical and decision-making requirements of competition. Consequently, manipulating task orientation allows practitioners to tailor training stimuli according to specific training objectives while maintaining an appropriate balance between physical, physiological, and tactical demands.

Beyond the differences observed between task orientations, an important practical consideration is how these training demands relate to competition requirements. While competition demands were not directly assessed in the present investigation, the observed differences between task orientations may provide useful insight into how training stimuli relate to match requirements. Previous research has shown that training loads do not always replicate competition demands and may either exceed or underestimate specific physical and physiological requirements depending on task design [18]. In this context, COM tasks may offer greater ecological validity due to their similarity to game situations, whereas SPE tasks appear to provide a stronger conditioning stimulus through higher physical and physiological demands. Therefore, an effective training process may require a balanced combination of both task types, allowing practitioners to simultaneously address competition-specific tactical requirements and the capacities needed to meet match demands.

Another important aspect examined in the present study was the influence of the court space within each task orientation, as spatial constraints are a critical factor in the design and regulation of training sessions. For instance, Sosa et al. [29] emphasized that the amount of space available during a task serves as a key variable for controlling exercise intensity, since manipulating the court space can effectively modulate the physical demands and physiological responses imposed on athletes. Likewise, Sansone et al. [47] reported that in longer-duration tasks conducted over larger spaces, players tend to self-regulate their effort to maintain efficiency, which can result in a lower average intensity per minute for some variables. The findings of the present investigation are consistent with the aforementioned observations, showing that expanding the court space generally leads to a significant increase in physical and physiological load across most analyzed variables. However, certain exceptions were noted, particularly in DIR tasks, for variables such as DIST, HACC, HSR, and PL, which likely reflect the specific structure and objectives of the training sessions analyzed.

Although the present study was conducted in a national-level women’s basketball team, the observed relationships between task orientation, court space, and training demands may also have relevance across different competitive standards. In elite international teams, where players are typically exposed to greater competition demands and training volumes, the magnitude of the responses observed in the present study may differ. However, the fundamental principles regarding the manipulation of task orientation and court space are likely to remain applicable. Conversely, in lower-level amateur teams, these findings may provide coaches with a practical basis for progressively increasing physical and physiological demands through simple modifications of task design, even when access to advanced monitoring technologies is limited. Therefore, while caution is warranted when extrapolating the absolute values reported in this study, the general training principles identified may be useful across a broad range of women’s basketball contexts.

While offering a deeper insight into the physical and physiological demands of women’s basketball training through LPS technology, this study is not without limitations. The sample size of 14 basketball players from a single team may limit the external validity of the findings. While the results may offer a valuable reference for comparable basketball settings, differences in training methodology, competitive environment, and player characteristics should be considered when interpreting their applicability to other populations. Future research including larger samples across different age groups and competitive levels would help strengthen the generalizability of these findings. Additionally, playing positions (centers, guards, and forwards) were not considered, and the use of convenience sampling may have reduced representativeness, although such constraints are common in applied sport settings. Despite these limitations, the study’s main strength lies in its novel integration of physical demand monitoring within a female basketball context, providing valuable methodological and practical insights. Therefore, future research should strive to include larger multi-team samples to enhance external validity and compare tasks of different task orientations but similar spatial demands to isolate the effects of opposition and tactical complexity. Incorporating positional analyses and contrasting training with competition demands would further align practice with real-game requirements. Overall, these efforts may help refine task-based training design and improve the ecological validity of performance monitoring in women’s basketball.

### 4.1 Conclusion

The findings of the present study indicate that both task orientation and spatial dimension significantly influence the physical and physiological demands placed on the athletes:1) DIR and SPE tasks demanded higher intensity than COM tasks; 2) In most cases, larger court spaces resulted in greater task demands and higher intensity; 3) In SPE and COM tasks, expanding the available court space was typically associated with a proportional rise in physical and physiological demands, whereas DIR tasks presented specific exceptions in which reduced court spaces elicited higher intensity.

### 4.2 Practical applications

Understanding how different tasks affect player load is essential for determining the most appropriate timing and structure of training sessions within the microcycle, with the dual objective of optimizing performance and minimizing injury risk. This information may assist coaches in distributing training stimuli more effectively across the weekly microcycle, ensuring that physical, physiological, and tactical objectives are appropriately aligned with the intended training load. Moreover, the integration of monitoring technologies, combining external and internal device-based sensors, may provide coaches with a powerful tool for quantifying these demands, enabling data-driven adjustments to task planning and design of training load, volume, and intensity.

The findings of this study provide practical guidance for optimizing training prescription in women’s basketball. Performance staff can strategically manipulate task orientation and court space to modulate training load. Although less cognitively demanding, DIR tasks still elicit substantial physical stress and are well suited for activation phases. In contrast, SPE tasks generate the highest conditional loads and are effective for developing high-intensity actions, though they require careful periodization to mitigate injury risk. On the other hand, COM tasks, remain essential for replicating the tactical and cognitive demands of match play. Furthermore, adjusting court space offers a simple yet effective strategy to regulate intensity, larger areas increase physical and physiological load, whereas smaller spaces emphasize contact, tactical decision-making, and cognitive engagement. Additionally, integrating LPS with device-based monitoring enhances the precision of load assessment, supporting individualized adjustments and more efficient management. These findings also underline the importance of accounting for sex-specific characteristics, as female players generally perform fewer high-intensity actions than males, reinforcing the need for targeted, high-demand stimuli within the microcycle. From a programming perspective, coaches may use SPE tasks, particularly when performed in larger court spaces, during high-load training days when the objective is to increase physical demands, including high-speed running, accelerations, and decelerations. Conversely, COM tasks may be prioritized when the primary objective is to reproduce the tactical and decision-making demands of competition while controlling excessive physical and physiological load. DIR tasks may be particularly useful during activation phases or technical-focused sessions, allowing coaches to maintain substantial physical stimulation with lower cognitive demands. Overall, this study provides an evidence-based framework to guide practitioners in designing training programs that enhance performance, accelerate adaptation, and minimize injury risk, even in settings with limited access to advanced technology.
